# Impact of Digital Interventions on the Treatment Burden of Patients With Chronic Conditions: Protocol for a Systematic Review

**DOI:** 10.2196/54833

**Published:** 2024-04-23

**Authors:** Manria Polus, Pantea Keikhosrokiani, Olli Korhonen, Woubshet Behutiye, Minna Isomursu

**Affiliations:** 1 Faculty of Information Technology and Electrical Engineering University of Oulu Oulu Finland; 2 Faculty of Medicine University of Oulu Oulu Finland

**Keywords:** chronic illness, treatment burden, eHealth, mHealth, digital health, mobile health

## Abstract

**Background:**

There is great potential for delivering cost-effective, quality health care for patients with chronic conditions through digital interventions. Managing chronic conditions often includes a substantial workload required for adhering to the treatment regimen and negative consequences on the patient’s function and well-being. This treatment burden affects adherence to treatment and disease outcomes. Digital interventions can potentially exacerbate the burden but also alleviate it.

**Objective:**

The objective of this review is to identify, summarize, and synthesize the evidence of how digital interventions impact the treatment burden of people with chronic conditions.

**Methods:**

The search, selection, and data synthesis processes were designed according to the PRISMA-P (Preferred Reporting Items for Systematic Review and Meta-Analysis Protocols) 2015. A systematic search was conducted on October 16, 2023, from databases PubMed, Scopus, Web of Science, ACM, PubMed Central, and CINAHL.

**Results:**

Preliminary searches have been conducted, and screening has been started. The review is expected to be completed in October 2024.

**Conclusions:**

As the number of patients with chronic conditions is increasing, it is essential to design new digital interventions for managing chronic conditions in a way that supports patients with their treatment burden. To the best of our knowledge, the proposed systematic review will be the first review that investigates the impact of digital interventions on the treatment burden of patients. The results of this review will contribute to the field of health informatics regarding knowledge of the treatment burden associated with digital interventions and practical implications for developing better digital health care for patients with chronic conditions.

**Trial Registration:**

PROSPERO CRD42023477605; https://www.crd.york.ac.uk/prospero/display_record.php?RecordID=477605

**International Registered Report Identifier (IRRID):**

DERR1-10.2196/54833

## Introduction

### Background

Digital technologies are now commonly used in daily life, bringing many new possibilities for connecting people and providing services. The use of mobile- and web-based digital health care interventions has increased during the COVID-19 pandemic and has been found to have high efficacy, accessibility, and cost-effectiveness in the self-management of chronic diseases [1-3].

As the global population is growing, the prevalence of chronic diseases has increased significantly [4]. The World Health Organization [4] has estimated that if this trend continues, by 2050 chronic diseases will be the cause of 86% of the 90 million deaths each year. This means a 90% increase in absolute numbers since 2019. Therefore, there is a continuous need for new interventions for the management of chronic diseases.

Chronic diseases often require regular long-term management, which requires patients to not only cope with their symptoms but also navigate services, interact with health professionals, and adhere to treatments, creating a significant burden for many patients [5]. Treatment burden is defined as both the workload required for self-management of disease and the impact treatment regimens have on the patient’s function and well-being [5]. Treatment burden can affect many domains: burden of taking medications, traveling to appointments, financial burden, impact on social life and emotions, and burden of accessing health care services [6,7]. A high treatment burden has been associated with poor adherence and worse disease outcomes [6].

The variety of available digital interventions can be tailored to meet the diverse needs of patients. For example, telemedicine and remote visits can reduce the need for traveling to medical appointments [8]. Mobile health apps, wearable technologies, and remote monitoring systems can track patients’ health data and alert health care professionals (HCPs) if intervention is required [9,10]. Electronic health records offer a central repository for patients’ history to minimize the paperwork and speed up the adherence process [11], while electronic prescription management improves patient safety as well as the efficiency and costs of prescribing medications [12]. Furthermore, patients’ portals, web-based support groups, and forums provide emotional and social support for patients [13].

The World Health Organization Classification of Digital Interventions, Services and Applications in Health [14] highlights a variety of digital health technologies for different types of services. In this study, we will focus on the point of service category of digital interventions. The point of service category includes those digital interventions that facilitate and deliver health care services to the patients, making it easier to see the connection between the treatment burden of the patient and the digital intervention. This category includes communication systems, community-based information systems, decision support systems, diagnostics information systems, electronic medical record systems, laboratory information systems, personal health records, pharmacy information systems, and a variety of telehealth systems. These digital interventions can include many different components, such as monitoring tools, decision aids, behavior change support, communication with HCPs, and web-based peer support groups.

Digital interventions may affect the treatment burden in multiple ways. With limited resources, digital interventions may be used to reduce the burden on the health care system, and staff end up offloading the burden to patients [15-18]. Patients may also find digital systems inaccessible or difficult to use [19] and struggle with digital stress [20]. However, digital interventions can expedite and simplify health care processes in a way that patients may receive treatment more efficiently, reducing the treatment burden for patients. For example, they can reduce the need for medical appointments and travel to hospitals [8] and make self-management easier and more motivating for patients [21,22].

Many systematic reviews have been conducted to investigate the treatment burden on patients with chronic conditions [6,7,23-26]. These reviews have provided insights into the definition, prevention, and patient’s experience of treatment burden. In addition, recent systematic and umbrella reviews about digital interventions have found that most digital interventions in health care are mobile- or computer-based [27-29]. The findings of the recent research are mostly focused on effectiveness, and the largest targeted condition group is mental illnesses [27,29]. However, we have observed a gap in the literature regarding systematic reviews combining these 3 concepts: treatment burden, digital interventions, and chronic conditions.

### Objective

The aim of this review is to identify gaps in the literature and summarize and synthesize currently available evidence of how digital interventions impact the treatment burden of people with chronic conditions. The impact can be a positive or negative effect on any domain of treatment burden. We aim to investigate if the results differ between chronic conditions with different levels of treatment burden or between interventions with different components.

### Research Questions

We have two primary research questions: (1) How can digital interventions impact the treatment burden on people with chronic conditions? (2) What kind of support can digital interventions provide for people with chronic conditions with their treatment burden?

## Methods

### Ethical Considerations

We followed the University of Oulu ethics process as defined in the guidelines from the Ethics Committee of Human Sciences [30]. According to the guidelines, an ethics board review is not needed for this protocol.

### Study Design

This protocol is reported according to the PRISMA-P (Preferred Reporting Items for Systematic Review and Meta-Analysis Protocols) 2015 [31]. We registered the protocol on the International Prospective Register of Systematic Reviews (PROSPERO CRD42023477605).

The systematic review will use a convergent design for systematic mixed studies reviews [32]. The mixed method approach was selected because qualitative results can help us to understand the phenomenon of treatment burden in the context of digital health care, and quantitative results can be used to generalize the qualitative findings by measuring their magnitude, trends, causes, and effects [33]. The convergent design was most suitable for this review since the research questions can be answered by both qualitative and quantitative findings.

### Information Sources and Search Strategy

A systematic search for papers published between January 1, 2013, and October 16, 2023, was conducted from bibliographical databases PubMed, Scopus, Web of Science, ACM, PubMed Central, and CINAHL. The following search string was used: (“Chronic illness” OR “chronic disease” OR “chronic diseases” OR “chronic illnesses” OR “chronically ill” OR “diabetes” OR “asthma” OR “cancer” OR “cystic fibrosis” OR “epilepsy” OR “rheumatoid arthritis” OR “HIV” OR “patient”) AND (“Digital” OR “Remote” OR “Mobile” OR “smartphone” OR “smartwatch” OR “smart ring” OR “smart device” OR “smart devices” OR “app” OR “mHealth” OR “eHealth” OR “web-based”) AND (“Treatment burden” OR “Burden of treatment” OR “Treatment impact” OR “Treatment workload” OR “Treatment inconvenience” OR “Treatment acceptability” OR “Illness burden” OR “Burden of illness” OR “Medication burden”). To identify relevant papers, a search strategy was conducted in an iterative way. The creation of the search strings for treatment burden was informed by previous systematic reviews on the topic [23,24]. Scoping searches were conducted in several potential databases focusing on health and biomedicine, information technology, nursing, psychology, or multiple disciplines (PubMed, Scopus, Web of Science, ACM, PubMed Central, CINAHL, IEEE, and APA PsycINFO). Scoping searches were conducted on October 15, 2023, in CINAHL and on October 12, 2023, in other databases. Searches conducted on IEEE and APA PsycINFO databases revealed no relevant results, so databases were excluded from the search strategy. Including only certain chronic conditions, for example, epilepsy or neurological conditions, was considered, but there was a limited number of studies found during the initial scoping searches. For example, in the scoping search for epilepsy conducted on October 12, 2023, only 3 relevant papers were identified. Therefore, we decided to keep the scope wide and include all chronic conditions in the search string.

The MeSH term “chronic disease” and search terms “coronary heart disease,” “heart disease,” “MS,” and “multiple sclerosis” were tested during a scoping search in PubMed, but they brought no new results and therefore were removed from the search terms. A supplementary search will be conducted from the citations contained in systematic literature reviews and scoping reviews that were found during the literature searches.

### Inclusion Criteria

We have included original publications written in English and accepted in peer-reviewed journals or conference proceedings. Qualitative, quantitative, and mixed method studies are included. The studies can be clinical trials, nonrandomized controlled trials, cross-sectional studies, longitudinal studies, observational studies, case studies, and other types of qualitative studies. Study design will be classified based on the tool from Grimes and Schulz [34]. Conference proceedings are included, but reviews, protocols, and book chapters are excluded.

We limited our search to publications after 2013 to include the last 10 years of research. Although digital health technology has developed quickly in recent years, the use of digital interventions in health care for chronic conditions goes farther than 10 years [35]. To the best of our knowledge, there are no previous systematic reviews relating to both digital health and treatment burden. However, studies before 2013 referred mostly to apps created only for research purposes, which were not available to the public at that time. Therefore, we decided to include papers published after 2013 to cover all relevant publications.

The study population in the included publications must consist of patients who have a chronic condition, their caregivers, or HCPs treating patients with chronic conditions. All ages and ethnicities are included. Only studies with outcomes regarding treatment burden for the patients are included. Studies that do not specifically mention the phrase “treatment burden” or “burden of treatment” but still discuss the impact of health care on the workload and burden for patients are also included. Only studies regarding a digital intervention that facilitates and delivers health care services to patients with chronic conditions are included.

### Selection of Studies

After the searches, all titles and abstracts from search results were uploaded to a web-based Covidence screening tool (Veritas Health Innovation), where duplicate records will be removed. All titles and abstracts were screened and selected for inclusion independently by 2 authors (MP and PK or OK). Full-text papers from the selected papers will also be screened and selected for inclusion independently by 2 authors (MP and PK or OK). Disagreements will be resolved by discussion.

### Data Extraction

Data on population characteristics, study design, aims, intervention characteristics, measures, and main results will be extracted using a predefined data extraction form in Covidence. Study design will be classified based on the tool from Grimes and Schulz [34]. Before proceeding with data extraction, MP will pilot the data extraction form with 5 papers to identify possible adjustment needs. Data extraction will be performed independently by 2 reviewers (MP and PK or OK). Disagreements will be resolved by discussion.

### Risk of Bias

The quality of the included studies will be assessed using Joanna Briggs Institute Critical Appraisal tools. Two reviewers (MP and PK or OK) will assess the quality of each included study independently. Based on the design of the eligible studies, we will use Joanna Briggs Institute checklists designed for randomized controlled trials, quasi-experimental studies, analytical cross-sectional studies, case-control studies, case series, and qualitative studies.

### Data Synthesis

A convergent integrated approach to synthesis and integration will be used [32]. This involves converting quantitative data into qualitative data followed by integration of the qualitative and quantitative evidence [36].

## Results

Currently, we have performed searches in the 6 selected databases, and 241 studies have been identified. Screening based on title and abstract excluded 192 studies. Overall, 69 studies have been included in the second round of study selection, which is ongoing. The review is expected to be completed in 2024.

## Discussion

This systematic review is performed to investigate the impact of digital health care on the treatment burden of patients with chronic conditions. This review is important because the world is currently facing increasing amounts of chronic diseases, and digital solutions are needed to improve the management of chronic diseases, which pose a significant burden on both health care systems and the patients themselves. However, it is essential to design the digital interventions in a way that helps patients to deal with their existing treatment burden and avoids further increasing the treatment burden. To the best of our knowledge, this will be the first review that covers the impact of digital health care on the treatment burden of patients with chronic conditions. The outcomes are expected to cover the positive and negative impacts of digital interventions on treatment burden and the different types of support digital interventions can provide to people with chronic conditions struggling with treatment burden. We aim to categorize different types of interventions and their components and find potential differences between interventions with different components and chronic conditions with different levels of treatment burden.

For the limitations of this review, the findings will depend on the number of eligible studies we will be able to identify and the quality of these studies. In addition, the studies identified for this review may be heterogeneous in terms of design, interventions, participant groups, and outcomes. Furthermore, our search will be restricted to peer-reviewed studies published in English.

## Abbreviations

HCP: health care professional
PRISMA-P: Preferred Reporting Items for Systematic Review and Meta-Analysis Protocols
